# Could tau-PET imaging contribute to a better understanding of the different patterns of clinical progression in Alzheimer’s disease? A 2-year longitudinal study

**DOI:** 10.1186/s13195-023-01237-2

**Published:** 2023-05-03

**Authors:** Julien Lagarde, Pauline Olivieri, Matteo Tonietto, Sébastian Rodrigo, Philippe Gervais, Fabien Caillé, Martin Moussion, Michel Bottlaender, Marie Sarazin

**Affiliations:** 1 Department of Neurology of Memory and Language, GHU Paris Psychiatrie & Neurosciences, Hôpital Sainte Anne, 75014 Paris, France; 2 Université Paris-Cité, 75006 Paris, France; 3 Université Paris-Saclay, BioMaps, Service Hospitalier Frédéric Joliot CEA, CNRS, 91401 Orsay, Inserm France; 4 Centre d’Evaluation Troubles Psychiques Et Vieillissement, GHU Paris Psychiatrie & Neurosciences, Hôpital Sainte Anne, Bâtiment Magnan, , 1 Rue Cabanis, 75014 Paris, France; 5 Université Paris-Saclay, UNIACT, Neurospin, Joliot Institute, CEA, 91140 Gif Sur Yvette, France

**Keywords:** Alzheimer’s disease, PET, MRI, Tauopathy

## Abstract

**Background:**

Monitoring the progression of Tau pathology makes it possible to study the clinical diversity of Alzheimer’s disease. In this 2-year longitudinal PET study, we aimed to determine the progression of [^18^F]-flortaucipir binding and of cortical atrophy, and their relationships with cognitive decline.

**Methods:**

Twenty-seven AD patients at the mild cognitive impairment/mild dementia stages and twelve amyloid-negative controls underwent a neuropsychological assessment, 3 T brain MRI, and [^18^F]-flortaucipir PET imaging (Tau1) and were monitored annually over 2 years with a second brain MRI and tau-PET imaging after 2 years (Tau2). We analyzed the progression of tau standardized uptake value ratio (SUVr) and grey matter atrophy both at the regional and voxelwise levels. We used mixed effects models to explore the relations between the progression of SUVr values, cortical atrophy, and cognitive decline.

**Results:**

We found an average longitudinal increase in tau SUVr values, except for the lateral temporoparietal cortex where the average SUVr values decreased. Individual analyses revealed distinct profiles of SUVr progression according to temporoparietal Tau1 uptake: high-Tau1 patients demonstrated an increase in SUVr values over time in the frontal lobe, but a decrease in the temporoparietal cortex and a rapid clinical decline, while low-Tau1 patients displayed an increase in SUVr values in all cortical regions and a slower clinical decline. Cognitive decline was strongly associated with the progression of regional cortical atrophy, but only weakly associated with SUVr progression.

**Conclusions:**

Despite a relatively small sample size, our results suggest that tau-PET imaging could identify patients with a potentially “more aggressive” clinical course characterized by high temporoparietal Tau1 SUVr values and a rapid clinical progression. In these patients, the paradoxical decrease in temporoparietal SUVr values over time could be due to the rapid transition to ghost tangles, for which the affinity of the radiotracer is lower. They could particularly benefit from future therapeutic trials, the neuroimaging outcome measures of which deserve to be discussed.

## Background

Alzheimer’s disease (AD) is a complex and heterogeneous disease, both in terms of clinical presentation [1] and evolution of cognitive disorders [2, 3]. Molecular imaging by positron emission tomography (PET) enables the in vivo detection of amyloid and tau pathologies, and the latter is closely associated with clinical symptoms in AD [4]. The prognostic value of tau PET imaging on the subsequent progression of brain atrophy and cognitive decline has been reported in recent studies [5–7], and regional tau radiotracer binding was able to predict cognitive decline in domain-specific brain areas [7]. Given the topographical information it provides, the longitudinal progression of [^18^F]-flortaucipir PET imaging could also be of interest to better understand the spatial and temporal relationships between tauopathy, cortical atrophy progression, which reflects neurodegeneration, and the cognitive decline over the same time interval. Longitudinal tau PET imaging studies are still relatively few [8]. The results published thus far do not seem to be completely consistent with some studies reporting an increase in the tau radiotracer binding over time in the temporal [9, 10], frontal [10–14], parietal [14], and occipital [13] regions or in all regions except the medial temporal lobes [15]. More surprisingly, some studies report a decrease in radiotracer uptake over time in some patients, which is occasionally considered a simple measurement error (processing artifacts, random statistical noise) [14–17]. Some studies reported a greater increase in radiotracer uptake over time when the binding is higher at baseline [14, 18], while others suggested the opposite relationship [17]. A greater increase in tracer uptake has also been reported in women and in younger patients [18]. In addition, the relationship between the evolution of tau radiotracer binding and cognitive evolution by considering specific cognitive domains has been little studied so far.

In the present study, we aimed to explore the longitudinal progression of the tau SUVr values and of cortical atrophy after a mean time interval of 2 years in a cohort of well-characterized AD patients at the mild cognitive impairment/mild dementia stages compared with controls, and their relationships with baseline regional tau load, and clinical decline in specific cognitive domains. We hypothesized that (a) tau SUVr values increase to varying degrees in AD patients depending on the cortical region considered, (b) tau-PET progression could be correlated with cortical atrophy progression, and (c) tau-PET progression could be associated with cognitive decline in specific domains.

## Methods

### Study design and participants

All participants were enrolled between March 2016 and November 2019 in the prospective longitudinal Shatau7-Imatau study (NCT02576821-EudraCT2015-000,257–20). The Ethics Committee (Comité de Protection des Personnes Ile-de-France VI) approved the study (Protocole n° 13–15). All subjects provided written informed consent.

Patients with AD at the mild cognitive impairment/mild dementia stages (*n* = 27) were included according to the following criteria: (i) cognitive phenotype of AD: temporoparietal cognitive deficit characterized by either a predominant amnestic syndrome (*n* = 18) or a predominant biparietal phenotype (*n* = 9); (ii) pathophysiological markers suggestive of AD, defined by both CSF AD profile (t-tau/Aß42 > 0.52) [19] and [^11^C]-PiB PET Global Cortical Index (GCI) > 1.45 [20, 21]; (iii) Clinical Dementia Rating (CDR) scale ≤ 1.

Twelve healthy elderly controls were included according to the following criteria: (i) Mini-Mental State Examination (MMSE) score ≥ 27/30; (ii) normal neuropsychological assessment; (iii) CDR = 0; (iv) no memory complaints; (v) negative PiB-PET imaging (GCI < 1.4).

At baseline, all participants underwent the same procedure including a complete clinical and neuropsychological assessment, 3 T brain MRI, [^11^C]-PiB, and [^18^F]-flortaucipir PET imaging (Tau1). Then, participants were monitored annually for 2 years with the same clinical and neuropsychological assessments as those performed at baseline and a second 3 T MRI and [^18^F]-flortaucipir PET imaging (Tau2) at the last visit.

### Functional and cognitive assessment

Standardized neurological and neuropsychological examinations were performed annually for 2 years, including the MMSE, CDR scale, and a standardized cognitive battery assessing verbal episodic memory, executive functions, gesture praxis, visuo-constructive functions, and language. As described in Lagarde et al. [7], we defined (a) a verbal episodic memory score, (b) an instrumental score, and (c) an executive score.

### Magnetic resonance imaging

All subjects underwent magnetic resonance imaging at the Centre de Neuro-Imagerie de Recherche (CENIR, ICM, Paris) using a 3 T whole-body PRISMA 64-channel system (Siemens) at inclusion and after two years of follow-up. The MRI examination included a three-dimensional (3D) T1-weighted volumetric magnetization-prepared rapid gradient echo (MP-RAGE) sequence (repetition time/echo time/flip angle: 2300 ms/3.43 ms/9°, inversion time = 900 ms, and voxel size: 1 × 1 × 1 mm^3^).

Baseline (MRI1) and 2-year (MRI2) MRIs were processed with the FreeSurfer 6.0.0 longitudinal processing stream (http://surfer.nmr.mgh.harvard.edu/) [22]. We considered the mean cortical thickness (CT) in the following VOIs: (i) the entorhinal cortex; (ii) a temporal meta-VOI composed of the entorhinal cortex, parahippocampus, fusiform gyri, inferior and middle temporal cortices [23]; (iii) a lateral temporal VOI; (iv) a lateral parietal VOI, (v) a medial parietal VOI, (vi) a frontal VOI, and (vii) an occipital VOI (as defined in Ossenkoppele et al.) [24]. In addition to the VOI analysis, MRIs were also processed with the pairwise longitudinal registration module of SPM12 to obtain annualized subtraction images (divided by the time interval between the 2 scans) for voxel-to-voxel analysis (http://www.fil.ion.ucl.ac.uk/spm). As performed in other works [25], we segmented the average T1 image obtained for each subject with the voxel-based morphometry pipeline implemented in the Computational Anatomy Toolbox (CAT12) and multiplied the grey matter in this image by the Jacobian difference map. The resulting image was subsequently normalized in MNI space and smoothed with an 8-mm full-width at half-maximum (FWHM) Gaussian kernel.

### [^11^C]-PiB and [^18^F]-flortaucipir PET imaging procedure

All subjects underwent [^11^C]-PiB and [^18^F]-flortaucipir PET (Tau1) and a second [^18^F]-flortaucipir PET exam (Tau2) after a mean delay of 2.3 ± 0.4 years. All PET examinations were performed at Service Hospitalier Frédéric Joliot (Orsay, CEA) on a high-resolution research tomograph (HRRT; CTI/Siemens Molecular Imaging). PET acquisitions were performed at least between 40 and 60 min after injection of 330.5 ± 63.6 MBq of [^11^C]-PiB and 80–100 min after injection of 382.0 ± 14.5 MBq of [^18^F]-flortaucipir for Tau1 and 368.2 ± 35.6 MBq of [^18^F]-flortaucipir for Tau2.

All corrections (attenuation, normalization, random and scatter coincidences) were incorporated in an iterative OSEM reconstruction. The partial volume effect (PVE) was corrected by directly modeling the detector spatial resolution properties (point spread function (PSF) modeling) in the image reconstruction algorithm [26, 27].

Parametric standard uptake value ratio (SUVr) images were created using BrainVisa software (http://brainvisa.info) on averaged images over 40–60 min after injection of [^11^C]-PiB and over 80–100 min after injection of [^18^F]-flortaucipir by dividing each voxel by the corresponding value for the eroded (2 mm) supra-tentorial white matter (segmented by Volbrain [28], which provided an accurate distinction of the basal ganglia from the white matter). This value of 2 mm is close to the intrinsic resolution of the HRRT PET camera and enables us to be far enough from the neighboring structures without reducing the volume of the region too much. We also performed analyses with the eroded (4 mm) cerebellar grey matter (to avoid including the superior part of the cerebellar vermis, which is a site of [^18^F]-flortaucipir off-target binding, and to avoid PVE from the CSF or occipital cortex) as a reference region, which was also used for PiB PET analyses as previously described [29, 30].

For the longitudinal tau-PET voxelwise analyses, we established annualized subtraction images, representing the annual change in SUVr values, by subtracting the images from the 2-year and baseline PET exams (Tau2 -Tau1) divided by the time interval between the two scans. To ensure the best alignment between the two PET images, we implemented a longitudinal pipeline as described in Schwarz et al. [30]. Briefly, we first created a linear template from the two PET exams using Advanced Normalization Tools (ANTs) software and then aligned each PET image (Tau1 and Tau2) to this template. We then created a nonlinear template from the two MRI scans of each subject and aligned the linear PET template to this non-linear MRI template, which was then normalized to MNI space. This allowed us to normalize each PET in the MNI space by combined transformations to perform the subtraction under optimized conditions. We individually assessed the quality of the registration of the Tau1 and Tau2 PET images in the 3-D space. The subtraction images were smoothed with an 8-mm FWHM Gaussian kernel.

The VOIs mentioned in the MRI section were aligned on the corresponding PET image in the native space by combined transformations with ANTs using the images and the registration matrices resulting from the construction of the templates described above. We extracted the mean SUVr values obtained with the different quantification methods (eroded supratentorial white matter or eroded cerebellar grey matter as reference regions) for each VOI, which were then subtracted (Tau2-Tau1). The values obtained were divided by the time interval between the 2 scans.

### [^18^F]-flortaucipir longitudinal analyses: Choice of the reference region

**Fig. 1:**
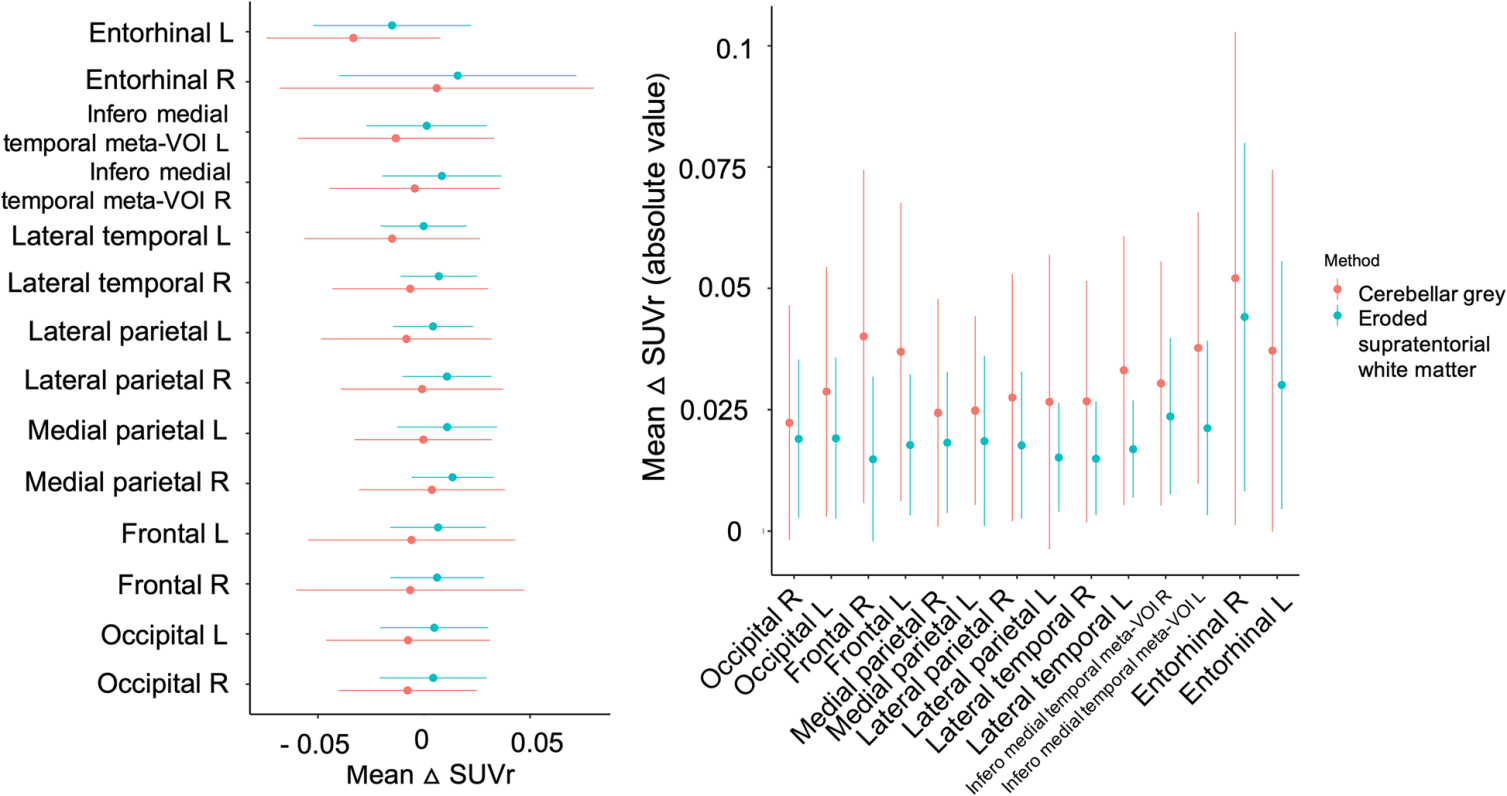
Progression of tau SUVr values in controls. Diagrams representing the average annualized differences in SUVr between the first and second tau PET exams (Tau2-Tau1) in amyloid negative control subjects (on the left), and the absolute value of this difference, representing the actual deviation from 0 (on the right) according to the quantification method used (eroded cerebellar grey matter and eroded supratentorial white matter serve as reference regions)

All the analyses in this study were performed using both cerebellar GM and eroded supratentorial WM as a reference region because in our population, we did not find any difference in baseline and Tau2-Tau1 SUV value in the cerebellar GM and in the eroded supratentorial WM between AD patients and controls. We found comparable results in terms of SUVr with the two reference regions. We chose to report only the results obtained with the supratentorial eroded WM reference region for the following reasons: (a) in controls, mean Tau2-Tau1 SUVr as well as its variability (standard deviation) were lower when using the supratentorial WM rather than the cerebellar GM (Fig. 1), and (b) previous works have highlighted the value of using the supratentorial eroded WM rather than the cerebellar GM as a reference region for the longitudinal analyses [29, 30] because it provides more consistent and repeatable estimates of flortaucipir change [12].

### Statistical analysis

#### Characteristics of the participants

The data were analyzed using R version 3.6.1 (R Core Team, 2017). Differences between subjects’ groups at baseline were assessed using analysis of covariance (ANCOVA), and the trajectories of cognitive decline and imaging progression over 2 years in AD patients and controls were compared using linear mixed effects models (interaction between the group, i.e., AD or control, and time, with subjects as a random intercept). Age and sex were included as covariates.

#### VOI analyses

ANCOVA or multiple regression analyses were used with age, sex, ApoE genotype, and the initial CDR sum of the boxes (CDR-SOB) as covariates and a Bonferroni correction. The level of statistical significance was set at *p* < 0.05.

#### Voxelwise analyses

We used VoxelStats 1.1 [31] to perform comparison and correlation analyses with a regression design with age, sex, ApoE genotype, and the initial CDR sum of the boxes (CDR-SOB) as covariates. The level of statistical significance was set at *p* < 0.05. A random field theory (RFT)-based multiple comparison correction [32] was performed with a clusterwise threshold of *p* < 0.001 and a cluster-forming threshold of *p* < 0.001.

#### Relationship between Tau2-Tau1 SUVr or cortical atrophy progression (MRI2-MRI1) and the cognitive decline in the AD patients

We used mixed effects models with the cognitive scores as dependent variables and age, sex, *APOE* genotype, and CDR-sum of boxes (SOB) as fixed effects. The model also included the interactions between the subtraction images and time, which were the regressors of interest. A subject effect was included as a random intercept in the model. Separate models were built for tau PET SUVr and cortical atrophy progression. These mixed effects models were run at the voxel level using VoxelStats 1.1.

## Results

### Characteristics of the participants

The subjects’ clinical, cognitive, and imaging characteristics at baseline and follow-up are summarized in Table 1. All AD patients had tau SUVr values in the inferomedial temporal meta-VOI greater than the control group mean plus 2 standard deviations.

**Table 1:** Main demographic, clinical, biological, and imaging characteristics at baseline and neuropsychological, tau-PET, and MRI data at 1 and 2 years (mean (SD))

			Baseline		One year		Two years	
			AD patients *n* = 27	Controls *n* = 12	AD patients *n* = 27	Controls *n* = 12	AD patients *n* = 27	Controls *n* = 12
***Demographic data***	Age (years)		68.6 (6.7)	68.7 (3.4)	-	-	-	-
	Sex (F/M)		14/13	9/3	-	-	-	-
	Education (years)		14.5 (4.4)	14.6 (3.3)	-	-	-	-
	Disease duration (years)		4.3 (3.6)	NA	-	-	-	-
	Cholinesterase inhibitors (*n*)		21	-	Introduced in 1, discontinued in 2	-	Introduced in 1	-
***Functional status***	CDR	0	0	12	0	12	0	12
		0.5	23	0	10	0	4	0
		1	4	0	17	0	16	0
		2	0	0	0	0	7	0
		3	0	0	0	0	0	0
	CDR sum of boxes		3.5 (1.8)	0	5 (2)	0	6.8 (2.7)	0
***Neuropsychological assessment***								
Global cognitive efficiency	MMSE (/30)		23.5 (3.1)^#^	28.8 (1.1)	21.6 (3.9)	29.3 (0.7)	18.8 (5.7)*	29.8 (0.5)
Memory score	FCSRT (free + total immediate and delayed recalls) (/128)		58.9 (27.4)^#^	108.8 (6.5)	43.9 (29.9)	110.4 (9)	28 (27.8)*	109.8 (5.3)
Instrumental score	(Naming + praxis + Rey figure copy) (/188)		169.8 (19.6)	185.1 (1.7)	160.2 (29.3)	185.8 (1.9)	145.6 (37.6)*	185.8 (2.7)
Executive score	(Digit spans + letter fluency 2 min + WAIS similarities)		43.4 (12.6)^#^	69.6 (7.9)	42.1 (15.4)	64.1 (11.1)	38.5 (18.8)	62.2 (11.3)
***Genetic status***	*ApoE* genotype (*n* with at least one E4 allele)		20	2	-	-	-	-
***CSF biomarkers***^***&***^	Amyloid peptide (pg/mL)		471.5 (113.7)	NA	-	-	-	-
	Total Tau (pg/mL)		640.5 (392.1)	NA	-	-	-	-
	Phospho-tau181 (pg/mL)		87.1 (37.1)	NA	-	-	-	-
***Molecular PET imaging***	PiB-PET SUVr (GCI)		2.89 (0.68)^#^	1.25 (0.08)	-	-	-	-
	Mean Tau-PET SUVr (infero-medial temporal meta-VOI)		1.57 (0.35)^#^	0.96 (0.08)	-	-	1.64 (0.25)	0.97 (0.06)
	Mean Tau-PET SUVr (lateral temporal VOI)		1.49 (0.33)^#^	0.97 (0.08)	-	-	1.53 (0.23)	0.98 (0.06)
	Mean Tau-PET SUVr (lateral parietal VOI)		1.51 (0.50)^#^	0.92 (0.07)	-	-	1.52 (0.38)	0.94 (0.07)
	Mean Tau-PET SUVr (medial parietal VOI)		1.52 (0.55)^#^	0.91 (0.09)	-	-	1.58 (0.46)	0.93 (0.06)
	Mean Tau-PET SUVr (frontal VOI)		1.17 (0.23)^#^	0.95 (0.07)			1.24 (0.26)	0.96 (0.08)
***MRI***	Fazekas score (0/1/2/3)		14/9/3/1	8/4/0/0	-	-	-	-
	Mean normalized HV		2.04 (0.30)^#^	2.41 (0.20)	-	-	1.9 (0.28)*	2.39 (0.25)
	Mean infero-medial temporal VOI CT (mm)		2.54 (0.18)^#^	2.86 (0.13)	-	-	2.38 (0.20)*	2.83 (0.14)
	Mean lateral temporal VOI CT (mm)		2.5 (0.14)^#^	2.78 (0.15)	-	-	2.35 (0.18)*	2.75 (0.15)
	Mean lateral parietal VOI CT (mm)		2.05 (0.20)^#^	2.35 (0.16)	-	-	1.96 (0.23)	2.31 (0.16)
	Mean medial parietal VOI CT (mm)		2.06 (0.19)^#^	2.33 (0.13)	-	-	1.98 (0.21)	2.3 (0.14)
	Mean frontal VOI CT (mm)		2.42 (0.13)	2.56 (0.11)	-	-	2.35 (0.14)	2.55 (0.11)

*CDR* Clinical Dementia Rating scale, *MMSE* Mini-Mental State Examination, *FCSRT* Free and Cued Selective Reminding Test, *WAIS* Wechsler Adult Intelligence Scale, *ApoE* apolipoprotein E, *CSF* cerebrospinal fluid, *GCI* Global Cortical Index, *SUVr* standardized uptake value ratio, *VOI* volume of interest, *HV* hippocampal volume (normalized to the intracranial volume), *CT* cortical thickness, *mm* millimeters

^*&*^Measured prior to inclusion in the study by enzyme-linked immunosorbent assay (ELISA) with INNOTEST assays (Fujirebio, Ghent, Belgium)

^#^*p* < 0.05 in the comparison with controls at baseline after Bonferroni correction for 18 tests

^*^p < 0.05 in the comparison of the 2-year trajectories between AD patients and controls after Bonferroni correction for 15 tests

### Tau2-Tau1 in AD patients and controls

#### VOI analysis

Within the AD group, the average SUVr values increased in each VOI between Tau1 and Tau2 (Table1) (6% increase in the inferomedial temporal meta-VOI and 6.7% increase in the frontal VOI over 2 years), except in the left temporoparietal region, where it decreased. SUVr values remained roughly stable over time in controls (1.3% increase in the temporal meta-VOI and 1.6% increase in the frontal VOI over 2 years). Compared to controls, Tau2-Tau1 in AD patients was increased in frontal regions (*p* = 0.0084 on the left and 0.018 on the right) but the difference did not resist correction for multiple comparisons.

#### Voxelwise analysis

**Fig. 2:**
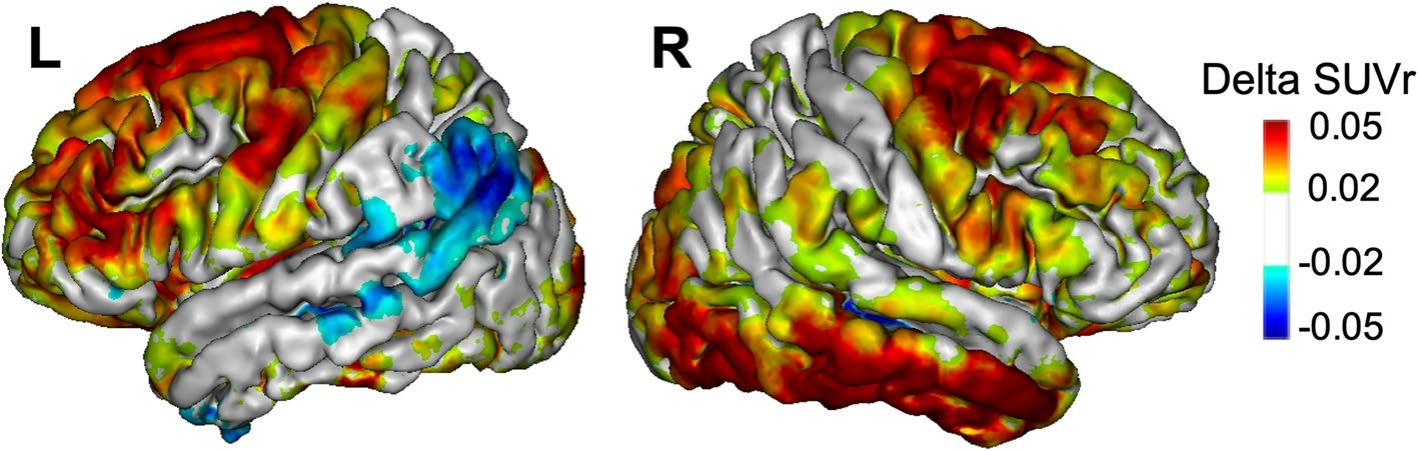
Progression of tau SUVr values in AD patients. Average annualized differences in SUVr between Tau1 and Tau2 (Tau2-Tau1) in AD patients (projection of the left (L) and right (R) hemispheres on the 3D MRI MNI template)

In the AD group, we found positive Tau2-Tau1 average SUVr values, especially in the bilateral frontal and right inferomedial temporal regions, in contrast with negative values in the parieto-temporal regions, especially on the left hemisphere (Fig. 2). In comparison to the controls, we found only a significant difference in Tau2-Tau1 values in a very circumscribed part of the left orbitofrontal cortex.

### Correlation between Tau1 and SUVr progression over time in AD patients

#### VOI analysis

**Fig. 3:**
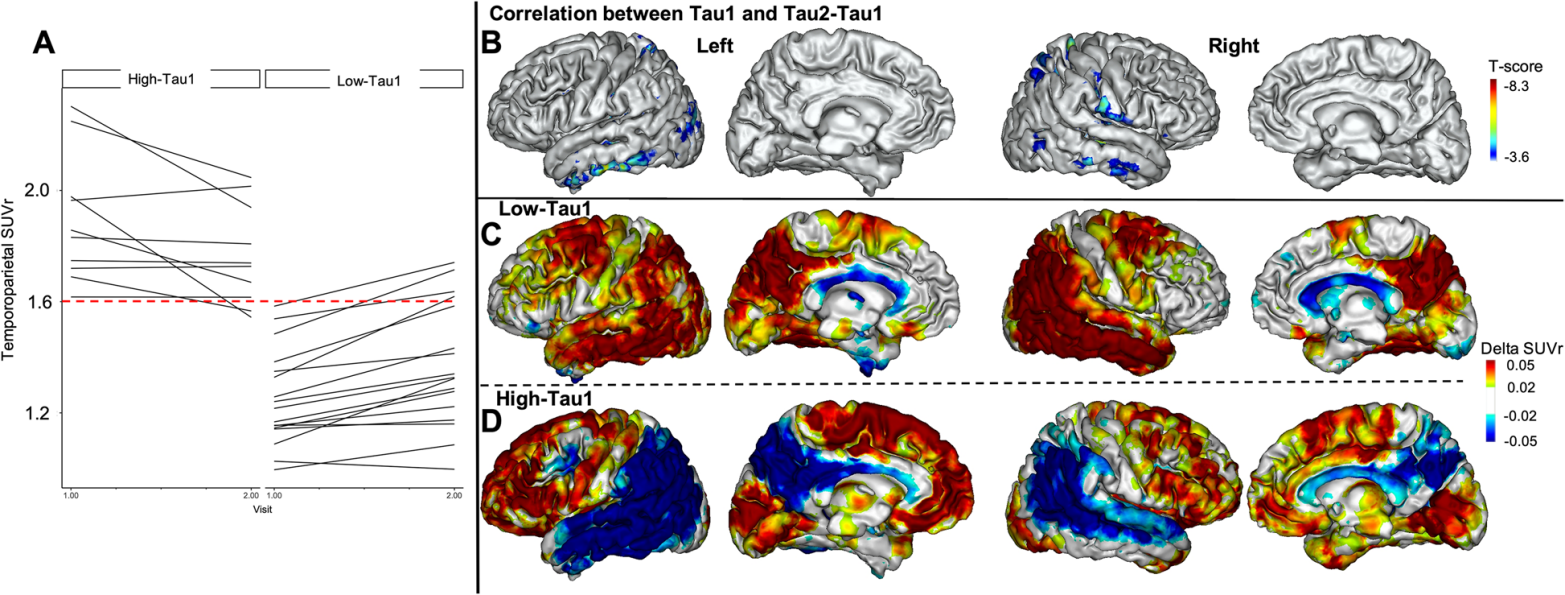
Tau SUVr progression in AD patients according to individual baseline tau tracer uptake. **A** Representation of the individual trajectories of the tau-PET SUVr in the temporoparietal meta-VOI in AD patients according to baseline SUVr values. **B** Results of the voxelwise correlation between baseline tau SUVr values (Tau1) and their progression over time (Tau2-Tau1) in AD patients with age, sex, ApoE genotype, and CDR-SOB as covariates. **C** Average annualized differences (Tau2-Tau1) in SUVr between the two tau PET exams in low-Tau1 AD patients. **D** Average annualized differences (Tau2-Tau1) in SUVr between the two tau PET exams in high-Tau1 AD patients (projection on the 3D MRI MNI template). RFT-based multiple comparison correction with a clusterwise threshold of *p* < 0.001 and a cluster-forming threshold of *p* < 0.001 in **B**

We found a significant negative correlation between Tau1 and Tau2-Tau1 in the temporal and lateral parietal cortices (*r*^2^ = 0.79, *p* = 0.0015 in the temporal meta-VOI, *r*^2^ = 0.83, *p* = 0.017 in the lateral temporal VOI and *r*^2^ = 0.7, *p* = 0.022 in the lateral parietal VOI). To analyze the relationship between Tau1 SUVr values and Tau2-Tau1 at an individual level, we defined a temporoparietal meta-VOI composed of the lateral temporal and lateral parietal VOIs. We observed two distinct profiles of tau progression according to Tau1 SUVr values (Fig. 3A). The receiver operating characteristic (ROC) analysis showed that Tau1 value could accurately discriminate patients with positive and negative Tau2-Tau1 values (AUC = 0.852) with an optimal cut-off value of 1.6. We have thus defined two sub-groups of patients according to their temporoparietal Tau1 value: “high-Tau1” AD patients (*n* = 10) and “low-Tau1” patients (*n* = 17). The main characteristics of high- and low-Tau1 AD patients are summarized in Table 2. Note that high-Tau1 patients are significantly younger than low-Tau1 patients.

**Table 2:** Main demographic, clinical, biological, and imaging characteristics at baseline and neuropsychological and imaging data at 1 and 2 years (mean (SD)) in the high- and low-Tau1 subgroups

			Baseline		One year		Two years	
			Low-Tau1 AD patients *n* = 17	High-Tau1 AD patients *n* = 10	Low-Tau1 AD patients *n* = 17	High-Tau1 AD patients *n* = 10	Low-Tau1 AD patients *n* = 17	High-Tau1 AD patients *n* = 10
***Demographic data***	Age (years)		72.2 (4.2)*	62.3 (5.3)	-	-	-	-
	Sex (F/M)		8/9	6/4	-	-	-	-
	Education (years)		14.4 (4.5)	14.8 (4.4)	-	-	-	-
	Disease duration (years)		5.4 (4.2)	2.6 (1.0)	-	-	-	-
***Functional status***	CDR	0	0	0	0	0	0	0
		0.5	16	7	7	3	4	0
		1	1	3	10	7	10	6
		2	0	0	0	0	3	4
	CDR sum of boxes		3.4 (1.6)	3.8 (2.1)	4.7 (2.2)	5.5 (1.5)	6.5 (2.9)	7.4 (2.5)
***Neuropsychological assessment***								
Global cognitive efficiency	MMSE (/30)		24.8 (3.1)	21.5 (2.2)	23.6 (3.2)	18.2 (2.3)	21.4 (4.9)#	13.9 (3.4)
Memory score	FCSRT (free + total immediate and delayed recalls) (/128)		47.6 (25.7)	78.1 (18.5)	38.9 (29.3)	52 (30.5)	26 (28.2)#	31.9 (28.5)
Instrumental score	(Naming + praxis + Rey figure copy) (/188)		180.2 (4.1)*	152.2 (23.2)	177.5 (9.0)	130.7 (28.2)	167.3 (20.4)#	104.7 (26.6)
Executive score	(Digit spans + letter fluency 2 min + WAIS similarities)		46.4 (13.2)	38.3 (10.1)	48.9 (14.0)	30.4 (9.7)	46.3 (18.1)#	23.7 (8.3)
***Genetic status***	*ApoE* genotype (*n* with at least one E4 allele)		16	4	-	-	-	-
***CSF biomarkers***	Amyloid peptide (pg/mL)		482.5 (116.4)	452.8 (112.4)	-	-	-	-
	Total Tau (pg/mL)		533.8 (176.7)	821.9 (574.9)	-	-	-	-
	Phospho-tau181 (pg/mL)		80 (27.3)	99.3 (48.8)	-	-	-	-
***Molecular PET imaging***	PiB-PET SUVr (GCI)		2.84 (0.64)	2.97 (0.76)				
	Mean Tau-PET SUVr (infero-medial temporal meta-VOI)		1.42 (0.19)	1.81 (0.43)			1.57 (0.20)#	1.75 (0.30)
	Mean Tau-PET SUVr (lateral temporal VOI)		1.32 (0.17)	1.77 (0.34)			1.44 (0.19)#	1.67 (0.23)
	Mean Tau-PET SUVr (lateral parietal VOI)		1.18 (0.21)*	2.1 (0.23)			1.31 (0.27)#	1.9 (0.19)
	Mean Tau-PET SUVr (medial parietal VOI)		1.21 (0.33)	2.1 (0.40)			1.34 (0.38)	1.97 (0.28)
	Mean Tau-PET SUVr (frontal VOI)		1.06 (0.12)	1.37 (0.23)			1.12 (0.17)	1.47 (0.23)
***MRI***	Fazekas score (0/1/2/3)		8/6/2/1	6/3/1/0				
	Mean normalized HV		1.97 (0.28)	2.14 (0.23)			1.85 (0.28)	1.97 (0.24)
	Mean infero-medial temporal VOI CT (mm)		2.57 (0.19)	2.5 (0.16)			2.45 (0.20)	2.26 (0.16)
	Mean lateral temporal VOI CT (mm)		2.52 (0.13)	2.47 (0.15)			2.41 (0.17)	2.24 (0.15)
	Mean lateral parietal VOI CT (mm)		2.15 (0.17)*	1.89 (0.14)			2.08 (0.17)	1.74 (0.12)
	Mean medial parietal VOI CT (mm)		2.14 (0.17)	1.93 (0.13)			2.11 (0.15)#	1.77 (0.12)
	Mean frontal VOI CT (mm)		2.39 (0.14)	2.47 (0.09)			2.36 (0.16)	2.35 (0.11)

*CDR* Clinical Dementia Rating scale, *MMSE* Mini-Mental State Examination, *FCSRT* Free and Cued Selective Reminding Test, *WAIS* Wechsler Adult Intelligence Scale, *ApoE* apolipoprotein E, *CSF* cerebrospinal fluid, *GCI* Global Cortical Index, *SUVr* standardized uptake value ratio, *VOI* volume of interest, *HV* hippocampal volume (normalized to the intracranial volume), *CT* cortical thickness, *mm* millimeters

^*^p < 0.05 in the comparison between groups after Bonferroni correction for 23 tests

^#^*p* < 0.05 in the comparison of the 2-year trajectories between low Tau1 and high Tau1 AD patients after Bonferroni correction for 16 tests

#### Voxelwise analysis

First, we analyzed the voxelwise correlation between Tau1 and Tau2-Tau1 in the whole AD group and confirmed that higher Tau1 values were associated with lower Tau2-Tau1 values in lateral temporal and parietal regions (Fig. 3B). Then, we studied the voxelwise average Tau2-Tau1 values in low- and high-Tau1 subgroups. We observed (a) an increase in the average SUVr values in the temporoparietal and frontal cortices in the low-Tau1 AD patients (Fig. 3C) with a significant difference from controls in the temporoparietal region and (b) an increase in the average SUVr values in the frontal lobes and a decrease in the temporoparietal cortices in the high-Tau1 AD patients (Fig. 3D), which were both significant compared to Tau2-Tau1 values in controls in the left hemisphere.

### Progression of cortical atrophy (MRI2-MRI1) in AD patients and controls

**Fig. 4:**
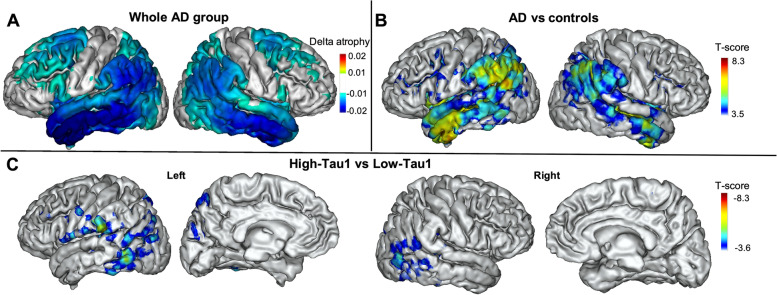
Progression of regional cortical atrophy. **A** Average annualized differences in grey matter volume between the two MRI scans in AD patients. **B** Results of the voxelwise comparisons of the longitudinal evolution of cortical atrophy between AD patients and controls. **C** Results of the voxelwise comparisons of the longitudinal evolution of cortical atrophy between high- and low-Tau1 AD subgroups with age and sex as covariates (projection on the 3D MRI MNI template). RFT-based multiple comparison correction with a clusterwise threshold of *p* < 0.001 and a cluster-forming threshold of *p* < 0.001 in **B** and **C**

Within the AD group, the average cortical atrophy progressed in the temporoparietal and frontal cortex (Fig. 4A). The comparison of atrophy progression between AD patients and controls showed significant differences in the temporal cortex (both on VOI and voxelwise analyses), including the postero-inferior part of the parietal cortex on the voxelwise analysis (Fig. 4B).

When we compared the low- and high-Tau1 AD subgroups, we found significantly higher atrophy progression in high-Tau1 than in low-Tau1 patients in the temporal and parietal lobes (Fig. 4C), without any difference in the hippocampal volume loss (Table 2).

### Correlation between tau and atrophy progression

In order to explore the relation between the progression of tau SUVr values and the progression of cortical atrophy, we studied the correlation between the tau-PET (Tau2-Tau1) and MR (MRI2-MRI1) subtraction images and did not find any significant result. The VOI analysis also did not yield significant results (*r*^2^ = 0.05 in the lateral parietal cortex, *r*^2^ = 0.11 in the lateral temporal cortex, *r*^2^ = 0.009 in the frontal cortex).

### Relationship between Tau2-Tau1 and cognitive decline

**Fig. 5:**
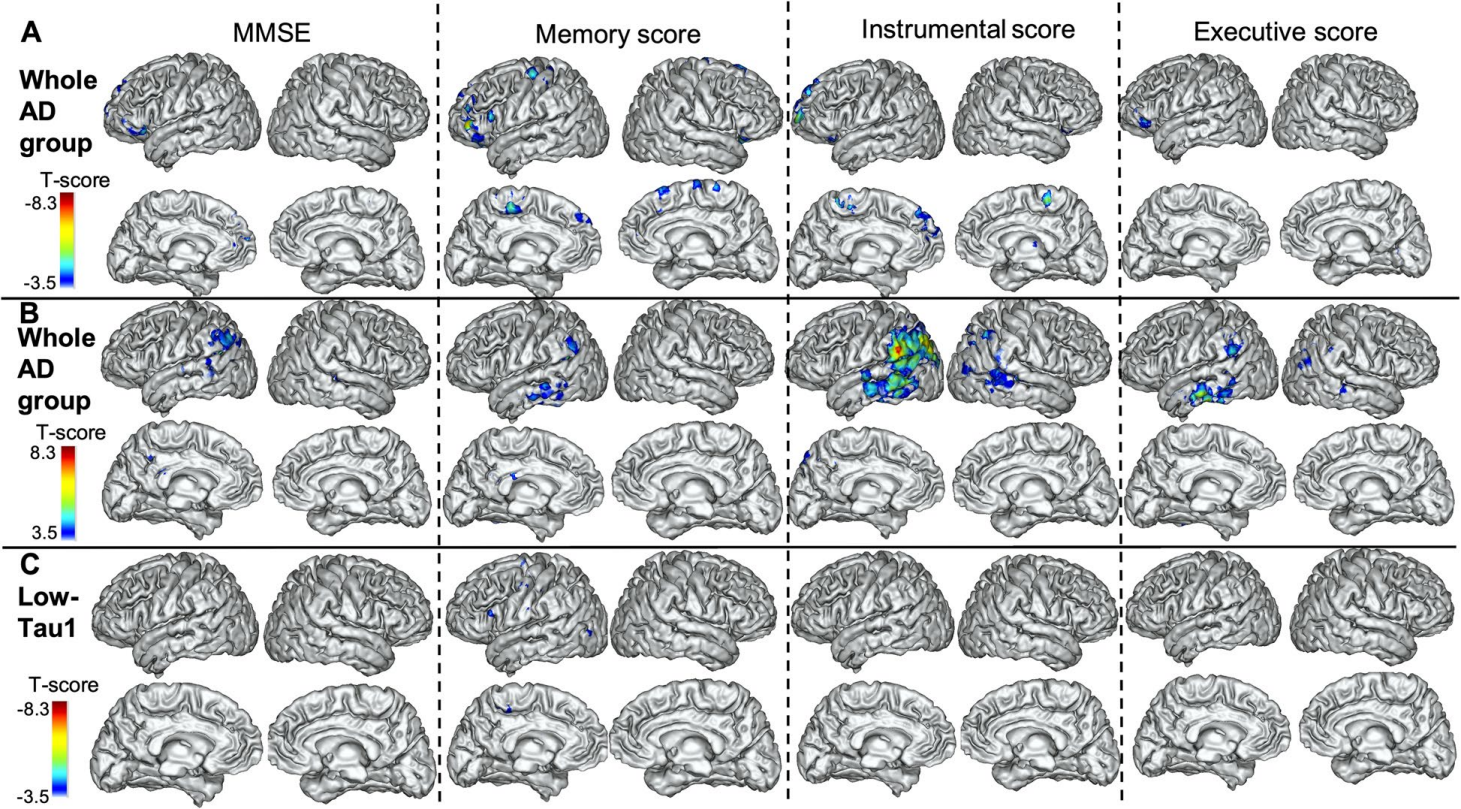
Relations of tau SUVr progression with cognitive decline. Results of the voxelwise analyses that show the association between the evolutions of the 4 cognitive outcomes over 2 years: MMSE, memory score, instrumental score and executive score and the evolution of the tau SUVr values **A**–**B** in the whole AD group (negative *t* values in **A**, representing the regions where the increase in SUVr values is associated with the decrease in cognitive scores, and positive *t* values in **B**, representing the regions where the decrease in SUVr values is associated with the decrease in cognitive scores), and **C** in the low-Tau1 AD subgroup (projection on the 3D MRI MNI template). RFT-based multiple comparison correction with a clusterwise threshold of *p* < 0.001 and a cluster-forming threshold of *p* < 0.001

Voxelwise analyses showed that the cognitive decline was associated with (a) an increase in tau SUVr in frontal regions (Fig. 5A) and (b) a decrease in tau SUVr in the temporoparietal region with a predominance on the left side regardless of the domain considered (Fig. 5B).

When we conducted a separated analysis in the subgroup of low-Tau1 AD patients, we found only a modest association between increased SUVr values in scattered areas predominantly in the left frontal lobe and the decline in the memory score (Fig. 5C). Note that low-Tau1 patients had a slower clinical progression than high-Tau1 patients (Table 2).

### Relationship between MRI2-MRI1 and cognitive decline

**Fig. 6:**
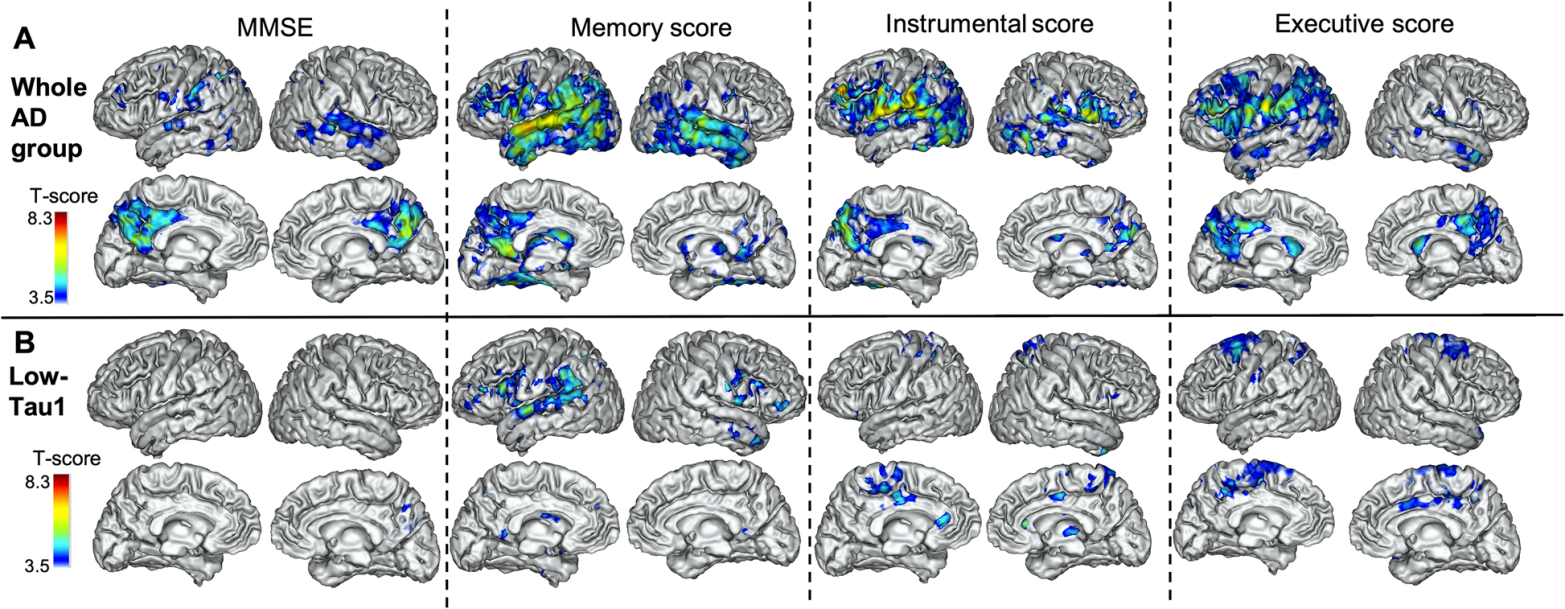
Relations of cortical atrophy progression with cognitive decline. Results of the voxelwise analyses that show the association between the evolutions of the 4 cognitive outcomes over 2 years: MMSE, memory score, instrumental score and executive score and the evolution of cortical atrophy **A** in the whole AD group and **B** in the low-Tau1 AD subgroup (projection on the 3D MRI MNI template). RFT-based multiple comparison correction with a clusterwise threshold of *p* < 0.001 and a cluster-forming threshold of *p* < 0.001

We found widespread positive associations (decreasing volume associated with decreasing cognitive scores) predominating in the temporal lobes for the memory score, the parietal regions for the instrumental score, and the fronto-parietal regions for the executive score (Fig. 6A).

The contrast between the different cognitive domains is more pronounced although the results are less strong when the low-Tau1 AD subgroup is considered separately (Fig. 6B).

## Discussion

The present study contributes to our understanding of the relationship among the progression of tau pathology as measured by [^18^F]-flortaucipir PET; the progression of the regional cortical atrophy, which is considered a marker of nonspecific neurodegeneration and neuronal loss; and cognitive decline in AD. We found in AD patients an expected increase in the average SUVr values over time in the frontal and right inferomedial temporal cortices, contrasting with a surprising decrease in the average SUVr values in the lateral temporoparietal cortex. Individual analyses revealed two distinct trajectories of tau SUVr values that depended on the initial intensity of temporoparietal tau load, leading to the definition of two AD subgroups called low- and high-Tau1. High-Tau1 patients, who were younger than their counterparts with low-Tau1, demonstrated an increase in SUVr values over time in the frontal lobe but also a decrease in the temporoparietal cortex and rapid clinical decline. In contrast, low-Tau1 patients displayed an increase in SUVr values over time in all cortical regions and a slower clinical decline.

Plateauing or decreasing tau SUVr values in some AD patients have been reported in previous studies and are most often attributed to measurement errors or artifacts [14–16]. However, it seems unlikely that our results are related to a measurement error, as we tried to minimize any source of error by using the most up-to-date techniques for the analysis of longitudinal tau PET data [29, 30]. We used a sophisticated method involving the construction of a nonlinear template of brain MRIs for registering the two PET images of each subject in MNI space and the VOIs defined from the segmentation performed by the FreeSurfer longitudinal pipeline to the PET images in the native space. This allowed us to optimize the quality of the VOI analyses and of the subtraction images used in the voxelwise analyses. In addition, we verified the consistency of our results regardless of the reference region (eroded supratentorial WM and cerebellar GM).

One can argue that the regional decrease in SUVr values could be an effect of regional cortical atrophy as brain atrophy progressed strongly in the regions where a decrease in SUVr values was observed, especially in patients with high Tau1. However, the following arguments go against this assumption: (a) we did not find a regional relationship between Tau2-Tau1 SUVr values and the progression of cortical atrophy when taking into account the effect of age, sex, ApoE genotype, and severity of the clinical alteration at baseline, which could have influenced the results; (b) we found an increase in SUVr values over time in regions where cortical atrophy also markedly progresses, such as the inferior temporal and frontal lobes; (c) we took into account the potential effect of atrophy in different ways, by verifying in subjects with a decrease in SUVr values over time that different grey matter segmentation (FreeSurfer and SPM toolbox) and partial volume effect correction methods (no partial volume correction, PSF correction within the reconstruction and GTM based method) did not modify the results obtained.

The decrease in SUVr values in temporoparietal regions could be related to a decrease of the tissue’s ability to retain pathologic aggregates over time or to the binding properties of the tau-PET radiotracer. It has been recently suggested that the decline in tau radiotracer binding may result from biological changes in the tau pathology that affect the affinity of the tracer [17]. Even if the time frame of this study is relatively short, this could be related to the transition to ghost tangles, which, contrary to mature tangles, contain predominantly 3R tau, for which the affinity of the radiotracer is lower, or to the occurrence of other modifications of the tau protein (post-translational modifications, truncation or conformational changes) which may affect the affinity of the tracer [33]. This decrease in SUVr values, regardless of whether it is related to brain atrophy, could therefore reflect the continuation of the disease process in the regions where it is most advanced and is ultimately more representative of the amplification of neuronal damage than the evolution of tau load.

Consistent with this view, we found a relationship between a decrease in SUVr values in the temporoparietal cortex and the severity of cognitive decline, particularly for the instrumental score. However, the opposite relation could have been expected in accordance with the well-known progression of tau pathology in AD. The finding of a significant association in the unexpected direction reinforces the idea that the decrease in SUVr values reflects the continuation of the disease process possibly due to the biological changes in tau pathology (transition to ghost tangles) such that the radiotracer is less able to detect at a more advanced stage of the disease.

We only found a weak relationship between the increase in SUVr values, mainly in the frontal regions, and cognitive decline, regardless of the cognitive domain considered. To ensure that this result was not biased by the regional decrease in SUVr values observed in some patients, we conducted the same analyses in the low Tau1 subgroup, in which SUVr values increase over time in all patients and found similar results. In addition, to account for the tendency of younger patients to have a higher Tau1 load and a biparietal clinical phenotype, age was included as a covariate in all analyses.

In contrast, we found a strong association between the progression of regional cortical atrophy and cognitive decline, depending on the cognitive component considered and congruent in anatomical-functional terms (memory and temporal lobes, instrumental functions and parietal cortex, and executive functions and fronto-parietal cortex), which is similar to what has been recently reported in the study of the predictive value of the baseline tau-PET [7]. The relationship between clinical progression and cortical atrophy progression seems, therefore, closer than that between cognitive decline and the evolution of tau-PET SUVr values, whereas the predictive value of baseline tau SUVr values on subsequent cognitive decline appears better than that of brain atrophy, as previously reported [7]. This finding is consistent with a previous study using the tau tracer THK-5317, which has the disadvantage of being strongly associated with MAO-B, especially in the basal ganglia. The results of this study suggested that changes in glucose metabolism over time, which is considered as a marker of neuronal injury, are more closely associated with clinical progression than changes in tau radiotracer binding [13].

## Limitations

The present study has some limitations. Its main weakness is the relatively limited number of AD patients included, which probably limits the statistical power and the generalizability of the results obtained, and could have influenced our voxelwise analyses. This limitation is inherent to longitudinal imaging studies in symptomatic AD patients. The relatively limited duration of follow-up, even if it is equal to or greater than most of the studies published to date, also constitutes a limitation of this study, in particular for the longer-term assessment of the impact of changes in tau protein, some of which may occur later.

## Conclusions

Beyond the utility of tau-PET for determining AD diagnosis and prognosis [7, 34], its use to follow the evolution of the disease and monitor the effect of possible treatments still raises certain issues. In order to treat AD at the earliest clinical stage, it is tempting to include patients with a relatively low initial tau load in clinical trials. When using such tau-PET imaging criteria, there is a risk of selecting patients who will have a slower clinical progression and thus decrease the ability to detect beneficial effects of treatments. Nevertheless, the inclusion of prodromal AD patients with a higher risk of rapid clinical progression characterized by an higher initial temporoparietal tau load and younger age will require great caution in interpreting the evolution of the SUVr values, which could be biased by a paradoxical decrease in some regions (potentially explained by a decrease of the tissue’s ability to retain pathologic aggregates over time or by a rapid transition to ghost tangles, for which the affinity of the tracer is lower). To avoid such difficulties, the choice of neuroimaging outcome measures deserves to be discussed, for example, by focusing on the frontal lobes where the SUVr values increase in all AD patients, and by also considering the progression of the regional cortical atrophy assessed by MRI.

## Data Availability

The datasets used and/or analyzed during the current study are available from the corresponding author on reasonable request.

## Abbreviations

AD: Alzheimer’s disease
PET: Positron emission tomography
CSF: Cerebrospinal fluid
GCI: Global Cortical Index
CDR: Clinical Dementia Rating
MMSE: Mini-Mental State Examination
MRI: Magnetic resonance imaging
VOI: Volume of interest
SPM12: Statistical Parametric Mapping 12
CAT12: Computational Anatomy Toolbox 12
FWHM: Full-width at half-maximum
PVE: Partial volume effect
SURr: Standard uptake value ratio
ANTs: Advanced Normalization Tools
GM: Grey matter
WM: White matter
RFT: Random field theory
AUC: Area under the curve
